# Spatial inequalities in breastfeeding initiation in the United States: A multiscale analysis of county-level determinants

**DOI:** 10.1371/journal.pgph.0005659

**Published:** 2026-07-15

**Authors:** Tony H. Grubesic, Wei Kang, Kelly M. Durbin, Edward Helderop

**Affiliations:** 1 School of Public Policy, University of California, Riverside, California, United States of America; 2 Childbirth International, Auckland, New Zealand; ICDDR: International Centre for Diarrhoeal Disease Research Bangladesh, BANGLADESH

## Abstract

Deepening our understanding of geographic variations in breastfeeding initiation (BFI) remains an important element in developing a comprehensive snapshot of maternal and infant health. Identifying spatial variations in socioeconomic, demographic, cultural, and environmental barriers to breastfeeding can advance intervention efforts to educate and encourage breastfeeding within a community. The purpose of this paper is to provide a comprehensive snapshot of county-level trends in BFI in the United States. Using a suite of exploratory spatial data analysis (ESDA) techniques and multiscale geographically weighted regression (MGWR), we highlight several important regional determinants of BFI and improve model performance, increasing the adjusted R^2^ from 0.641 in a traditional OLS model, to 0.852 in the MGWR model. We concluded the paper by identifying potential locations for public health interventions, especially in Appalachia and the Gulf Coast regions of the United States, and provide three relatively cost-effective strategies to improve BFI that focus on community support, healthcare infrastructure, and education.

## 1. Introduction

Breastfeeding provides numerous health benefits for both the mother and the child [1]. For example, research suggests that breastfeeding mothers may see reductions in breast and ovarian cancers, as well as postpartum weight loss [1,2]. Breastfed children may have improved cognitive function [3], a lower occurrence of dental malocclusions [4], and protection from Type II diabetes [5,6] and obesity (Hernández Cordero & Pérez Escamilla, 2022). When combined with the short-term benefits of reduced infant mortality (Sankar et al., 2015) and fewer occurrences of sudden infant death syndrome [7,8], appropriately, the Healthy People 2030 program in the United States seeks to increase the proportion of exclusively breastfed infants through six months of age to 42.4% (currently 27.9% as of 2022), and any breastfeeding through twelve months to 54.1% (currently 40.8% as of 2022) [9,10].

A core challenge for increasing breastfeeding initiation (BFI) in the United States is that the country’s social, demographic, economic, cultural, and environmental fabric is highly variable, and many national-level summary statistics tracked by programs such as Healthy People 2030 mask substantial geographic variation in breastfeeding rates at lower levels. For example, during the 2018–2019 reporting period, BFI rates by county varied from 22% to more than 90% across the United States [11]. As a result, to deepen our understanding of community-level drivers of BFI, it is imperative that public health officials consider the use of statistical models that can account for these spatial heterogeneities, and multiscale geographically weighted regression (MGWR) [12,13] is a promising approach.

Again, many factors drive vast differences in BFI at the local level, and in numerous instances, spatial heterogeneities in breastfeeding intersect with both race and socioeconomic status (SES). Consider, for example, a recent study that leveraged data from the 2016–2019 Pregnancy Risk Assessment Monitoring System (PRAMS), 88% of women reported initiating breastfeeding, and information from family and friends and from breastfeeding support groups (e.g., La Leche League) was consistently associated with breastfeeding duration across most racial and ethnic groups. However, the effects were consistently smaller among Black and Hispanic women (versus white women), with over 50% of American Indian and ~25% of Black women reporting no breastfeeding initiation or stopping breastfeeding because of concerns regarding a return to school or work [14]. Further, it is important to note that these racial groups do not exhibit a uniform spatial distribution in the United States [15].

One critical undercurrent to the social determinants of breastfeeding is the *availability* of community support for breastfeeding mothers. Support takes many forms, from Baby-Friendly Hospitals [16,17], to organizations such as La Leche League, Breastfeeding USA [18], the Women Infants and Children (WIC) program [19], local experts (e.g., International Board Certified Lactation Consultants) [20–22], and telelactation support [23,24]. Much like the local variability in breastfeeding initiation rates, there are also substantial heterogeneities in the availability of community support options for mothers across the United States [25]. As a result, issues of access and equity to these support resources remain a concern throughout the U.S. [26].

These spatial variations in BFI and support resources also intersect with race, SES, and breastfeeding duration [27]. Again, the results suggest that white mothers breastfed longer, on average, than Black mothers, with mothers in professional/managerial work breastfeeding longer than those in more service-oriented positions [27]. Socioeconomic status also influences BFI and duration. Recent work suggests that fewer lower-SES women continued breastfeeding for six months compared to higher-SES women (51% versus 70%, respectively) [28]. For more details, Standish and Parker[29] provide a thorough overview of the social determinants of breastfeeding in the United States and note that future interventions to address issues of breastfeeding initiation and continuation should occur at the policy, *community*, organization, and individual levels. This call for action is important, but it highlights a particularly challenging problem: *which communities*? After all, recent research suggests that interventions can work, especially for communities of color [30], but identifying the specific communities (beyond race and income) that need interventions remains challenging.

The purpose of this paper is to help identify the gaps in breastfeeding initiation in the United States and their socio-demographic drivers. Again, this is a challenging task because BFI data can be difficult to wrangle and integrate with reliable demographic and socioeconomic data. Further, given the need to identify specific communities ripe for intervention or alternative public health policies, one must also use a suite of spatial statistical methods that account for the spatial interdependencies of BFI data and its socio-demographic drivers across multiple scales.

## 2. Subject and Methods

### 2.1. Study design and location

This cross-sectional, ecological study provides an exploratory spatial analysis of breastfeeding initiation at the county level for the United States. The U.S. offers an interesting setting for examining the variability in breastfeeding initiation and its potential demographic and socioeconomic drivers. With a population of ~340 million residents, recent estimates suggest that ~75.5 million women of reproductive age lived in the United States in 2020, and that approximately 3.9% of these women were pregnant at any point in time [31]. Of course, given the substantial demographic and socioeconomic diversity in the U.S., the pregnancy (and breastfeeding) experience of these women is highly variable. 2020 Census results indicate that approximately 59.3% of U.S. residents are White, 18.9% Hispanic and Latino, 12.6% Black, and 5.9% Asian [32]. Finally, recent CDC estimates (2024) indicate that the percentage of babies who were ever breastfed increased from 83.2% in 2015 to 85.7% in 2022.

### 2.2. Data collection

We obtained county-level breastfeeding initiation data from the CDC and the National Immunization Survey [11]. Breastfeeding initiation was defined as receiving any breast milk or colostrum between delivery and discharge from the birth facility (or birth certificate for home births). Notably, this definition differs from the WHO’s definition of early initiation of breastfeeding (EIBF), which specifies breastfeeding within the first hour of birth (WHO, 2024). We adopt the CDC definition here because it is the standard metric used in national U.S. breastfeeding surveillance and is consistently applied across all counties in the dataset. The summary initiation data are reported at the county or county-equivalent level (i.e., when cities function as their own counties), using the mother’s place of residence as the geographic reference. These data include all infants born during 2018 or 2019 except for infants who: 1) were transferred to another facility within 24 hours of delivery, 2) died before birth certificate completion, 3) are missing breastfeeding initiation data, or 4) are born to foreign residents or residents of California, Michigan, Alaska, Hawaii, American Samoa, or the U.S. Virgin Islands.

We combined the CDC data with 2019 Newborn Screening test data from California [33] and harmonized them with the CDC data. These data represent county-level aggregates on the percentage of babies that were breastfed during their hospital stay. Unfortunately, Michigan data proved too onerous to process because the state reports data using local agency geographies, which can range from a single county to an agglomeration of counties. Ultimately, we are left with 3,011 county-level observations for the lower 48 contiguous states (minus Michigan) in the United States. This represents 95.8% of all counties in the 50 states and DC, and 92.8% of all counties if one includes U.S. territories. Importantly, these data use information from birth certificates recorded at the time of hospital discharge, rather than from parental recall surveys. This contemporaneous recording eliminates recall bias as a concern for the breastfeeding initiation measure used in this study.

The exclusion of Michigan leaves 83 counties missing from our analysis of the contiguous United States. While this exclusion is unfortunate, a cartographic analysis of Michigan suggests it shares many of the same BFI characteristics as its neighboring states - Ohio, Wisconsin, Illinois, and Indiana. Specifically, Michigan’s BFI rate (67.03%) falls below the national average (78.77%), but the aggregate state rate masks substantial variation across its agglomerated reporting districts, which range from 53.93% in the Detroit area (urbanized and majority Black population) to 84.72% in the Western Upper Peninsula (rural and largely white population) [34]. In short, while we cannot directly model the BFI outcomes in Michigan, we believe the results of our study are generalizable, and excluding Michigan will not introduce any systematic biases.

We obtained demographic and socioeconomic data from the U.S. Census Bureau, drawing upon the American Community Survey (ACS) 5-year estimates for 2013–2017. The use of the 2013–2017 ACS 5-year estimates was intentional to ensure temporal ordering between explanatory variables and the 2018–2019 breastfeeding initiation rates. By using socioeconomic characteristics measured prior to the outcome period, we reduce concerns about simultaneity and strengthen the interpretation of these variables as pre-existing contextual conditions. Table 1 illustrates the descriptive statistics for our dependent variable and the demographic and socioeconomic indicators. The use of all the secondary data in this study did not require ethical approval.

**Table 1:** Descriptive Statistics for Demographic and Socio-economic Indicators for U.S. Counties.

	*Description*	*Data source*	*Mean*	*Standard deviation*
***Dependent Variable***				
*bf*	Breastfeeding initiation rate		78.77	12.28
***Independent Variables***				
*Edu*	% of bachelor’s degree or higher for Population 25 years and over	ACS Data Profile	21.18	9.3
*UrPop10*	% of population that lives in urban areas (2010 census)	2010 Census Urban Areas	41.74	31.48
*Black*	% of non-Hispanic Black Population	ACS Detailed Table	9.16	14.66
*Hispanic*	% of Hispanic Population	ACS Detailed Table	9.26	13.84
*Asian*	% of Asian Population	ACS Detailed Table	1.24	2.32
*Disability*	% of Population With a disability	ACS Data Profile	15.89	4.4
*FemHead*	% of female-headed households with children	ACS Data Profile	6.11	2.36
*Stable10*	% of population moved in before 2010	ACS Data Profile	63.66	7.13
*Pop*	Total Population	ACS Subject Tables	102593.8	331994.8
*SelfEmp*	% of self-employed	ACS Data Profile	7.77	3.87
*Occupation*	% of labor force working in Management, business, science, and arts occupations	ACS Data Profile	31.56	6.53
*Income*	Median household income for 2013–2017 (in 2017 dollar)	ACS Detailed Table	49632.84	13124.67
*IncGrowC*	Income Compound Annual Growth Rate (2012–2017)	ACS Detailed Table (2012, 2017)	0.33	1.73
*Assis*	% of households receiving cash public assistance income	ACS Detailed Table	2.34	1.66
*Gini*	Gini index for spatial income inequality (Gini on tract-level median household income for each county)	ACS Detailed Table (tract-level)	0.1	0.07
*FemLaborF*	% of female (16 years and over) labor force participation	ACS Subject Tables	54.11	7.06

### 2.3. Variable Selection and Processing

Building on the theoretical and operational/methodological findings from previous studies and given data availability, we used the county-level breastfeeding initiation rate as our dependent variable [26,35,36]. All independent variables were standardized (mean = 0, standard deviation = 1), following the recommendations from a series of multiscale geographically weighted regression (MGWR) studies [13,37,38]. This standardization makes the interpretation of optimal bandwidths from MGWR more intuitive, functioning as indicators of the spatial scales of the underlying processes. As a result of this standardization, one should interpret the coefficient estimates as the change in the breastfeeding initiation rate associated with a one-standard-deviation change in the corresponding independent variable.

### 2.4. Data Analysis

This study used a combination of exploratory spatial data analysis (ESDA) [39] and geovisualization approaches to determine spatial variability in breastfeeding initiation at the community level in the United States. In particular, we used MGWR [38] to measure the geographic scales over which different demographic and socioeconomic drivers of the breastfeeding initiation process may operate. Compared with an OLS model, which assumes a global, constant association between each independent variable and the dependent variable, MGWR models these associations as spatially varying by operationalizing Tobler’s first law of geography: “Everything is related to everything else, but near things are more related than distant things” [40]. Specifically, we use only geographically close observations in the calibration of the local regression model for each county, with closer observations assigned larger weights.

Additionally, compared with its precursor, geographically weighted regression (GWR) [41], or an alternative, such as a Bayesian spatial regression model [42], MGWR does not assume that all of the underlying processes operate at the same spatial scale (i.e., all regional, all local, or all global), MGWR allows for different processes to operate at different spatial scales – some of which may be highly local, while others are more regional or global in nature [13,37]. Ultimately, MGWR produces a unique (optimized) bandwidth for each relationship (e.g., education and breastfeeding initiation) in the model, which serves as an indicator of the spatial scale at which each relationship operates [37,43]. More formally, consider the following formal expression of MGWR:


$$\begin{array}{c} y_{i}=\beta_{\text{0}}\left(u_{i},v_{i}\right)+\sum_{j=\text{1}}^{k}\beta_{bwj}\left(u_{i},v_{i}\right)x_{ij}+\varepsilon_{i} \end{array}$$ (1)

where $y_{i}$ is the dependent variable (i.e., breastfeeding initiation) for location i, $x_{i}$ is the value of the j th independent variable for location i, $\left(u_{i},v_{i}\right)$ represent the geographic coordinates of location i, $\beta_{\text{0}}\left(u_{i},v_{i}\right)$ is the intercept for location i, $\beta_{bwj}\left(u_{i},v_{i}\right)$ is the local regression coefficient for the jth independent variable at location i, bwj is the optimal bandwidth for $\beta_{j}$, and $\varepsilon_{i}$ is the residual error for location i. We used the adaptive bisquare kernel to define spatial weights used in MGWR:


$$\begin{array}{c} w_{ijh}=\left\{ \begin{array}{c} {\left[\text{1}-{\left[\frac{d_{ih}}{G_{ij}}\right]}^{\text{2}}\right]}^{\text{2}},\text{ if}\;d_{ih}<G_{ij}\\ \text{0},\;\text{Otherwise} \end{array}\right. \end{array}$$ (2)

where $w_{ijh}$ is the weight of the h th observation in the estimation of the j th variable at location i, the optimal bandwidth for $\beta_{j}$ is the number of nearest observations (bwj) used for the calibration of each local coefficient, $G_{ij}\;$is the distance from the location i to its bwjth nearest neighbors, and $d_{ih}$ is the distance from location i to location h. As indicated in Equation (2), only counties that are among bwjth nearest neighbors to the focal unit are used in the calibration for $\beta_{j}$ at location i and their contributions decline as they are farther away. MGWR is operationalized as a general additive model (GAM) and uses an iterative back-fitting approach to calibrate a series of GWR models based on their partial residuals until the MGWR model converges on an optimal solution [44]. Inference for local coefficients was conducted by deriving local standard errors and applying multiple testing-adjusted critical t-values based on the effective number of parameters (ENP) for each covariate, thereby reducing false positives in the interpretation of local coefficients [43].

In plain terms, MGWR can be thought of as running a separate regression model for each county, with the influence of nearby counties weighted more heavily than that of distant ones. The ‘neighborhood’ used to estimate each local relationship is allowed vary in size: some demographic or socioeconomic drivers may operate consistently across the entire country, resulting in a large neighborhood (or bandwidth) where all observations contribute to the local estimation (effectively equivalent to a global OLS model), while other relationships may be more localized and vary more rapidly across space, leading to a smaller bandwidth in which only nearby observations are used for the local estimation. This flexibility makes MGWR especially well-suited for this type of study, where the social, economic, and cultural factors shaping breastfeeding initiation are unlikely to operate uniformly across such a large and diverse country.

## 3. Results

### 3.1. Gaussian Model

**Fig 1:**
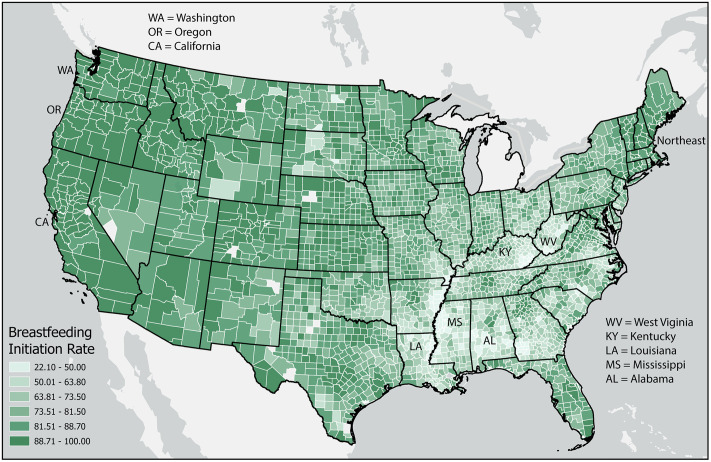
Breastfeeding initiation rate (%) by county, for 2018-2019 in the contiguous United States, excepting Michigan. Basemap Sources: Contains information from OpenStreetMap and OpenStreetMap Foundation, which is made available under the Open Database License.

Fig 1 highlights the spatial distribution of breastfeeding initiation rates for the lower 48 contiguous states (minus Michigan) for 2018–2019. Please note that we created all maps using publicly available data, including county/county-equivalents (Census, 2025) and basemap layers (OSM, 2025). There are several patterns worth highlighting. First, the Northeast (e.g., Vermont, New Hampshire, Massachusetts) and portions of the West Coast (California, Oregon, Washington) exhibit some of the highest BFI rates in the country. However, many Gulf Coast states (e.g., Louisiana, Mississippi, Alabama) and portions of Appalachia (e.g., West Virginia, Kentucky) have much lower BFI rates.

To better understand these variations in BFI and to provide some perspective on the advantages of MGWR, we first estimated a conventional global Gaussian model as a benchmark. We report these results in Table 2. These results support what much of the public health research community already understands about breastfeeding initiation in the United States. Specifically, BFI is significantly and negatively associated with the proportion of Black residents, the share of female-headed households, county income growth, the proportion of the population with disabilities, and population turnover. Conversely, higher BFI rates are positively associated with more educated and affluent communities, as well as high rates of self-employment and of women in the labor force. The model also reveals a significant, positive relationship between the proportion of the Hispanic population and BFI. At the same time, diagnostic tests indicate important limitations of the global model. For instance, the Breusch–Pagan test (BP = 379.493, *p* < 0.001) indicates heteroskedasticity in the residuals and the residuals exhibit substantial positive spatial autocorrelation (Moran’s I = 0.4067, pseudo *p* = 0.001, based on 999 permutations and *k*-nearest-neighbor spatial weight matrix with *k* = 10), suggesting that the global model does not adequately capture the spatial structure in the data. Therefore, while the global model offers a coherent summary of the socio-demographic determinants of county-level BFI, these diagnostics indicate that its assumptions are not fully satisfied. Additionally, it explains only 64.1% of the variance and assumes spatial stationarity in all associations. This assumption limits its ability to capture the localized social, economic, and institutional dynamics that may shape breastfeeding behavior differently across regions. To address this limitation, we next employ MGWR, which allows both the strength and scale of associations to vary across space, thereby offering a more nuanced understanding of the spatial processes shaping breastfeeding initiation.

**Table 2:** Global OLS Model Results.

Model type		Gaussian		
Number of Observations		3011		
Number of Covariates		17		
Global Regression Results				
Residual sum of squares:		162156.820		
Log-Likelihood:		-10273.786		
AIC:		20581.572		
AICc:		20583.801		
BIC:		138174.797		
R2:		0.643		
Adj. R2:		0.641		
Breusch–Pagan		379.493 (*p* < 0.001)		
Moran’s I		0.4067 (*p* = 0.001)		
*Variable*	*Est.*	*SE*	*t(Est/SE)*	*p-value*
Intercept	*78.118*	*0.248*	*314.966*	*0.000*
Edu	*3.084*	*0.315*	*9.786*	*0.000*
UrPop10	*-0.332*	*0.255*	*-1.301*	*0.193*
Black	*-3.003*	*0.190*	*-15.795*	*0.000*
Hispanic	*2.190*	*0.164*	*13.368*	*0.000*
Asian	*-0.304*	*0.191*	*-1.593*	*0.111*
Disability	*-1.157*	*0.221*	*-5.231*	*0.000*
FemHead	*-2.922*	*0.214*	*-13.675*	*0.000*
Stable10	*-2.105*	*0.194*	*-10.874*	*0.000*
Pop	*0.506*	*0.166*	*3.053*	*0.002*
SelfEmp	*2.212*	*0.185*	*11.970*	*0.000*
Occupation	*-1.428*	*0.266*	*-5.371*	*0.000*
Income	*2.257*	*0.265*	*8.534*	*0.000*
IncGrowC	*-0.830*	*0.142*	*-5.846*	*0.000*
Assis	*0.278*	*0.089*	*3.115*	*0.002*
Gini	*-0.460*	*0.229*	*-2.011*	*0.044*
FemLaborF	*1.363*	*0.219*	*6.214*	*0.000*

### 3.2. MGWR Model

Table 3 reports key MGWR diagnostics, including the optimal bandwidth for each conditional relationship (indicating its spatial scale), the ENP, and the adjusted critical *t*-values used for local statistical inference. Of note is the improved adjusted coefficient of determination value of 0.852 when compared to the Gaussian model (Adjusted R^2^ = 0.641) and a reduced AICc value of 18405.8 for the MGWR (versus 20583.8 in the OLS). Additionally, in contrast to the global model, the MGWR residuals do not exhibit statistically significant spatial autocorrelation (Moran’s I = 0.0014, pseudo p = 0.409, based on 999 permutations), suggesting that the MGWR has effectively accounted for the spatial structure in the BFI pattern. In short, the MGWR is a substantially better-fitting model than the global OLS model with the same set of dependent and independent variables, owing to its flexibility in modeling local, regional, and global relationships. For instance, educational attainment and urban population both have a bandwidth of 3010, indicating that the model uses all counties in each local estimation and that these relationships operate at a global scale. In contrast, the association between disability and BFI varies more substantially across space, with an optimal bandwidth of 49. Table 4 highlights the differing levels of spatial heterogeneity and presents summary statistics for the MGWR local coefficient estimates. While the local coefficients for educational attainment are uniformly positive and exhibit very little variation (standard deviation = 0.016), the coefficients for disability are predominantly negative but include some positive values, with substantially greater dispersion (standard deviation = 2.097).

**Table 3:** Multi-Scale Geographically Weighted Regression (MGWR) Results.

Spatial kernel:			Adaptive bisquare	
Criterion for optimal bandwidth:			AICc	
Score of Change (SOC) type:			Smoothing f	
Termination criterion for MGWR:			1.00E-05	
*MGWR Bandwidths*				
*Variable*	*Bandwidth*	*ENP_j*	*Adj t-val (95%)*	*Adj alpha (95%)*
Intercept	43	176.85	3.635	0.000
Edu	3010	1.203	2.039	0.042
UrPop10	3010	1.212	2.042	0.041
Black	3010	1.027	1.972	0.049
Hispanic	2042	2.008	2.244	0.025
Asian	3010	1.613	2.158	0.031
Disability	49	143.82	3.581	0.000
FemHead	144	47.912	3.282	0.001
Stable10	1471	3.878	2.488	0.013
Pop	886	7.256	2.704	0.007
SelfEmp	893	5.902	2.634	0.008
Occupation	3010	1.246	2.053	0.040
Income	1538	2.809	2.371	0.018
IncGrowC	2605	2.168	2.274	0.023
Assis	1550	2.382	2.309	0.021
Gini	3010	2.049	2.049	0.041
FemLaborF	797	2.612	2.612	0.009
*Diagnostics*				
Residual sum of squares			58132.461	
Effective number of parameters (trace(S)):			408.051	
Degree of freedom (n - trace(S))			2602.949	
Sigma estimate:			4.726	
Log-likelihood:			-8729.384	
AIC:			18276.870	
AICc:			18405.847	
BIC:			20735.277	
Moran’s I			0.0014 (*p* = 0.409)	
R2			0.872	
Adjusted R2			0.852	

**Table 4:** Summary Statistics for MGWR Parameter Estimates.

*Variable*	*Mean*	*STD*	*Min*	*Median*	*Max*
Intercept	80.321	5.145	65.177	80.5	93.968
Edu	1.615	0.016	1.558	1.618	1.643
UrPop10	-0.850	0.004	-0.853	-0.851	-0.838
Black	-2.593	0.014	-2.619	-2.59	-2.562
Hispanic	0.980	0.637	0.264	0.844	1.893
Asian	-0.397	0.016	0.450	-0.392	-0.370
Disability	-0.429	2.097	-10.294	-0.364	8.174
FemHead	-1.240	1.098	-4.066	-1.208	1.583
Stable10	-1.283	0.362	-1.943	-1.263	-0.721
Pop	1.283	0.966	0.035	0.976	3.797
SelfEmp	1.166	0.665	0.013	1.032	2.503
Occupation	-0.251	0.017	-0.280	-0.254	-0.220
Income	2.226	0.386	1.177	2.315	2.704
IncGrowC	-0.416	0.111	-0.586	-0.457	-0.192
Assis	-0.121	0.127	-0.349	-0.116	0.086
Gini	-0.297	0.033	-0.353	-0.307	-0.220
FemLaborF	1.408	0.893	-0.255	1.43	2.609

### 3.3. Global Trends for Breastfeeding Initiation

**Fig 2:**
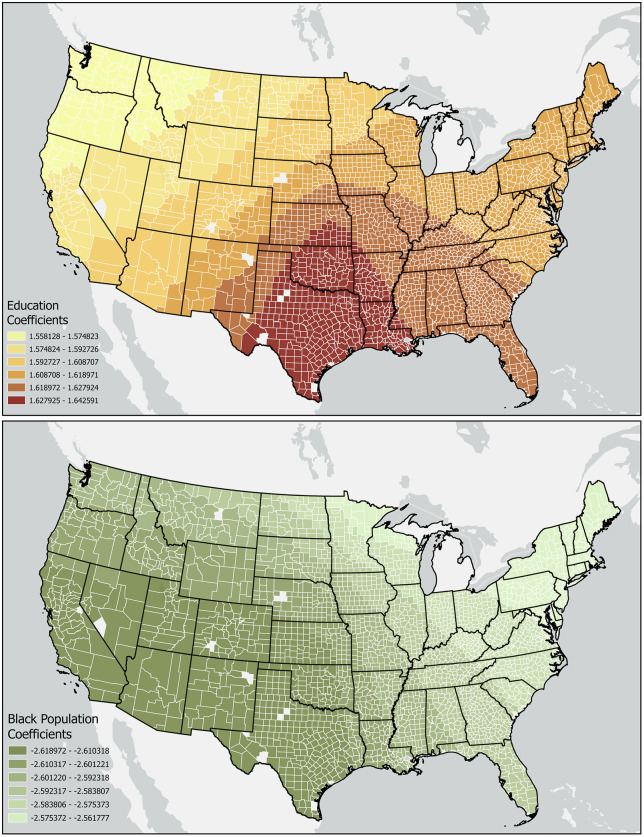
MGWR local coefficient estimates for education (a) and Black population proportion (b) in relation to county-level breastfeeding initiation rates across the contiguous United States, excepting Michigan. Basemap Sources: Contains information from OpenStreetMap and OpenStreetMap Foundation, which is made available under the Open Database License.

One simple way to parse the results for the MGWR is to examine the associations between BFI and each independent variable at their respective operational scales. For example, at the global level, our measures for education, non-Hispanic Black population share, Asian population share, and urban population share all display optimal bandwidths of 3010 – suggesting global relationships. While there is some geographic variation in education, with southern counties exhibiting a slightly stronger association than western counties, this variation is relatively small (Fig 2A, Table 4). Thus, on average, a one-standard-deviation increase in the percentage with a bachelor’s degree or higher is associated with a 1.62 percentage-point increase in BFI. Similarly, the MGWR model identifies a statistically significant but substantively weak global negative association between urban population share and BFI. Across all counties, a one-standard-deviation increase in the share of residents living in urban areas (31.48 percentage points) is associated with only a 0.85 percentage-point decrease in breastfeeding initiation. While the relationship is spatially stable and consistently negative, its small magnitude suggests that urbanization has a limited standalone influence on breastfeeding initiation.

The opposite occurs for the Black population measure. Specifically, a one-standard-deviation increase in Black population share (14.7 percentage points) is associated with a 2.56-2.62 percentage-point decline in breastfeeding initiation (Fig 2B). For the Asian population measure, we see a similar trend, especially in the Central and Western U.S. Specifically, a one standard deviation increase (2.32 percentage points) was associated with a 0.37–0.45 percentage point decrease in BFI. Counties in the eastern U.S. showed no statistically significant relationship.

Finally, the conditional relationships between professional/managerial occupation share and BFI, and between the Gini coefficient and BFI, operate at a global scale. However, local coefficient estimates for both are never statistically significant at the 5% level after multiple testing correction. Thus, while the share of the professional workforce and the Gini coefficient showed a negative, statistically significant relationship with breastfeeding initiation in the global OLS model, the MGWR results suggest otherwise: neither showed a statistically significant relationship.

### 3.4. Regional trends in breastfeeding initiation

**Fig 3:**
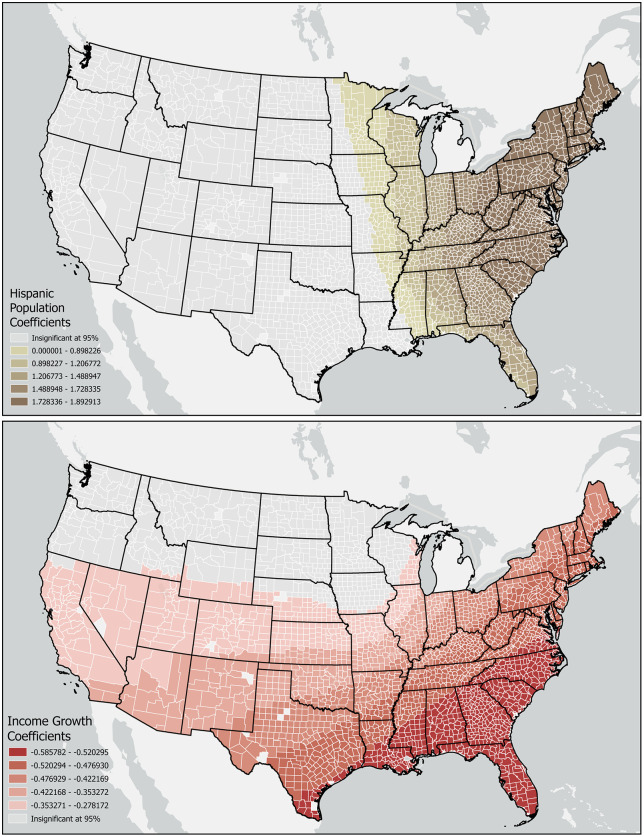
MGWR local coefficient estimates for Hispanic population proportion (a) and income growth (b) in relation to county-level breastfeeding initiation rates across the contiguous United States, excepting Michigan. Basemap Sources: Contains information from OpenStreetMap and OpenStreetMap Foundation, which is made available under the Open Database License.

The MGWR model revealed an optimal bandwidth of 2,042 counties for the percent Hispanic population variable, suggesting a broadly positive association between Hispanic population share and BFI. As displayed in Fig 3A, there are substantial effects concentrated in the Northeast, Mid-Atlantic, and parts of the Southeast. In these areas, coefficients range from 1.85 to 1.89, indicating that a one-standard-deviation increase in the Hispanic population (13.84 percentage points) is associated with a 1.9 percentage-point increase in BFI. The relatively large bandwidth suggests limited local variation. Even in the West, which exhibits high Hispanic population shares, there is no significant relationship, suggesting that contextual factors may attenuate the effect.

The MGWR model reveals a widespread, statistically significant negative association between income growth and breastfeeding initiation, with an optimal bandwidth of 2,605. The strongest effects are concentrated in the Southeast (Fig 3B). In these counties, the coefficient ranges from –0.59 to –0.53, indicating that a one-standard-deviation increase in the income growth rate (1.73 percentage points) is associated with a 0.53-0.59 percentage-point decrease in BFI. This negative relationship gradually diminishes moving north and west, where it weakens and eventually becomes statistically insignificant. Given this negative association for the Southwest, it is important to reiterate that we are measuring income growth, not income. All racial groups in the United States (including the Southeast) benefitted from income growth between 2012 and 2017, but the Southeast also corresponds to the Black Belt [45] (i.e., larger Black population), so these results are not surprising, and neither is the decay in significance as one moves north and west.

There are additional regional effects for our measure of residential stability (optimal bandwidth = 1,471). The results suggest that the MGWR reveals a statistically significant negative association between residential stability and BFI, indicating that a one standard deviation increase in the percentage of the population who moved in before 2010 (7.13 percentage points) is associated with a decrease of up to 1.94 percentage points in breastfeeding initiation rates. The association is strongest in the Southeast and gradually weakens westward, although all counties remain significant. We observe a similar regional effect for median income, which is consistently positive but shows varied associations with BFI across regions. Specifically, a one-standard-deviation increase in median household income ($13,125) is associated with up to a 2.7 percentage-point increase in breastfeeding initiation.

Finally, the MGWR model identifies a negative, regional relationship between public assistance and breastfeeding initiation (optimal bandwidth = 1,550). The most substantial effects (–0.35 to –0.32) concentrate in the Northern Plains states, including North Dakota and South Dakota, eastern Montana, central Wyoming, and western Nebraska. In these areas, a one-standard-deviation increase in public assistance rates (1.66 percentage points) corresponds to a 0.32–0.35 percentage-point decline in BFI. Additional moderate but significant effects (–0.25 to –0.32) are observed across the West Coast, Upper Midwest, and parts of the Mountain West, while most of the South and East show no statistically significant associations.

### 3.5. Smaller Regional and Local Trends in Breastfeeding Initiation

While global and regional trends in breastfeeding initiation are informative, the smaller regional and local variation between communities is often the most difficult to identify and understand. Nevertheless, deepening our understanding of these smaller regional and local trends is often the most crucial information for developing strategies to enhance breastfeeding education, community support, and, ultimately, BFI. The MGWR model identified five independent variables with relatively smaller regional and local effects on BFI: population, self-employment, female labor force participation, disability, and female-headed households with children.

The variables of population, self-employment, and female labor force operate at about the same geographic scale, with optimal bandwidths of 886, 893, and 797, respectively. While these scales may not seem particularly local, it is important to recall that the regional variables detailed above had bandwidths about twice this size.

**Fig 4:**
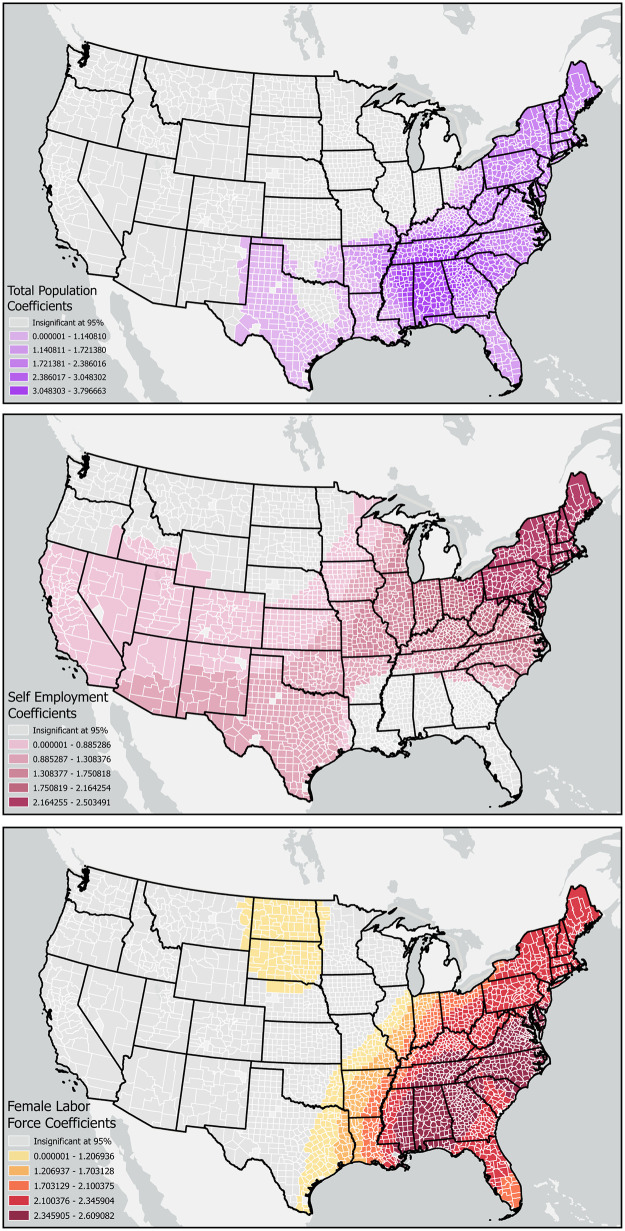
MGWR local coefficient estimates for total population (a), share of self-employment (b), and female labor force participation (c) in relation to county-level breastfeeding initiation rates across the contiguous United States, excepting Michigan. Basemap Sources: Contains information from OpenStreetMap and OpenStreetMap Foundation, which is made available under the Open Database License.

The MGWR model reveals a statistically significant positive association between total population (bandwidth = 886) and breastfeeding initiation rates, concentrated across the Southeast and parts of Appalachia (Fig 4A). Coefficients in these areas range from 2.58 to 3.80, indicating that a one-standard-deviation increase in population size (332,000) is associated with up to a 3.8 percentage-point increase in BFI. This relationship weakens and becomes statistically insignificant in much of the western U.S. and parts of the Midwest. Similarly, there was a significant positive association between the county-level share of self-employed individuals and breastfeeding initiation across much of the eastern United States, with coefficients reaching 2.5. This result implies that a one-standard-deviation increase in self-employment (3.87 percentage points) is associated with up to a 2.5 percentage-point increase in breastfeeding initiation. However, this relationship gradually weakens moving westward and southward, with several regions – particularly across the Southwest, Rockies, and Deep South – showing no statistically significant effect (Fig 4B).

Finally, the MGWR model identified a strong positive relationship between female labor force participation and breastfeeding initiation (Fig 4C). Statistically significant effects are concentrated in the Southeast and Mid-Atlantic, where a one-standard-deviation increase (7.06 percentage points) in female labor force participation is associated with a 0.76 to 2.61 percentage-point increase in BFI. The strongest associations are found in Alabama, Georgia, Tennessee, North Carolina, Virginia, and surrounding states, suggesting that greater female workforce participation may correspond with higher awareness or support for breastfeeding in these areas.

**Fig 5:**
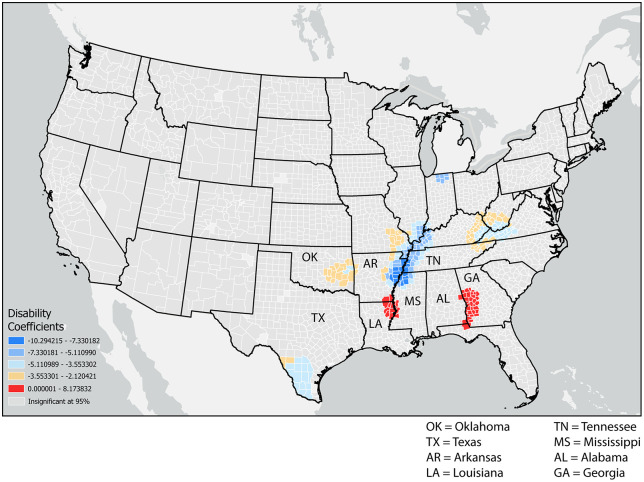
MGWR local coefficient estimate for disability prevalence in relation to county-level breastfeeding initiation rates across the contiguous United States, excepting Michigan. Basemap Sources: Contains information from OpenStreetMap and OpenStreetMap Foundation, which is made available under the Open Database License.

The disability variable in the MGWR model revealed a strongly localized and spatially heterogeneous association between disability prevalence and breastfeeding initiation (Fig 5). For example, statistically significant negative associations dominate the spatial pattern, particularly in three distinct clusters: (1) a large region encompassing portions of Arkansas, Tennessee, Missouri, Illinois, and Kentucky; 2) a cluster in Virginia; and 3) a cluster in Oklahoma and parts of South Texas. In these areas, a one standard deviation increase in disability prevalence (4.4 percentage points) is associated with declines in breastfeeding initiation ranging from –2.12 to –10.29 percentage points, suggesting substantial disparities where disability-related barriers may be more pronounced.

In contrast, positive associations emerge in two smaller county clusters: one along the border of Alabama and Georgia, and the other spanning eastern Arkansas, northeastern Louisiana, and western Mississippi (Fig 5). In these regions, higher disability prevalence is associated with increases in breastfeeding initiation of up to 8.2 percentage points, indicating that local health programs or community-level resilience factors may offset the challenges typically faced by individuals with disabilities. It is important to note that the disability measure used here is the ACS aggregate ‘any disability’ variable, which combines cognitive, ambulatory, vision, hearing, self-care, and independent living difficulties into a single measure. As a result, it is not possible to determine whether specific disability types drive the contrasting regional associations observed.

The small bandwidth (43) confirms that this variable operates at a fine-grained spatial scale, underscoring the need for localized interventions tailored to community-specific conditions. These results underscore the critical importance of place-based maternal and child health policies that recognize the intersection between disability prevalence and community support for breastfeeding.

**Fig 6:**
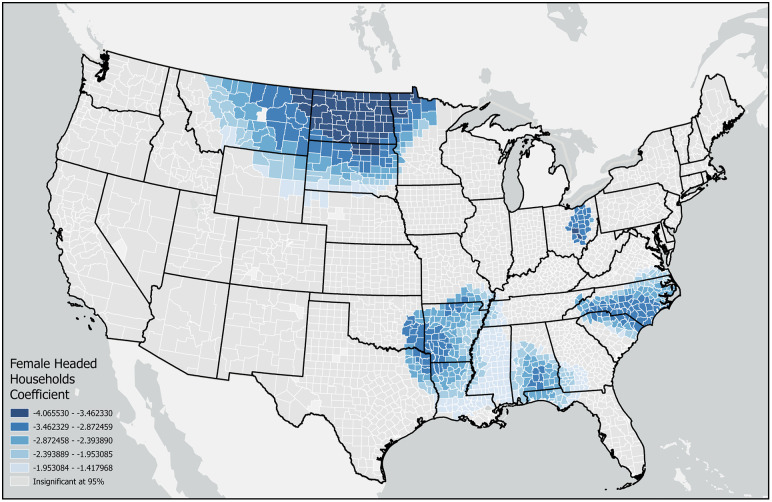
MGWR local coefficient estimate for the proportion of female-headed households in relation to county-level breastfeeding initiation rates across the contiguous United States, excepting Michigan. Basemap Sources: Contains information from OpenStreetMap and OpenStreetMap Foundation, which is made available under the Open Database License.

Lastly, the MGWR results reveal a spatially heterogeneous relationship between the percentage of female-headed households with children and breastfeeding initiation rates (Fig 6). Specifically, female-headed households are associated with a stronger, more negative, and more localized effect on breastfeeding initiation. As a result, statistically significant negative associations were observed in some regions, including counties across the Upper Midwest, Mississippi Delta, Appalachia, and Eastern North Carolina. These patterns suggest that higher rates of female-headed households are linked to lower breastfeeding initiation rates in these areas. The optimal bandwidth of 144 further underscores that this relationship operates at a localized spatial scale, reinforcing the importance of regional context when analyzing the influence of family structure on maternal and child health behaviors.

Beyond these somewhat obvious interpretations, it is critical to note that these significant relationships were found in counties with *both* low and high levels of female-headed households, suggesting that the strength of the relationship does not depend solely on prevalence. For instance, in North Dakota and parts of northern Montana and Minnesota, a 2.36 standard deviation increase in the percentage of female-headed households with children is associated with a 3.12 to 4.07 percentage point decrease in breastfeeding initiation rates. In contrast, no statistically significant association was observed in much of the West, the Northeast, and the Midwest, highlighting substantial spatial variation in this relationship across the U.S.

### 3.6. A Final Perspective on MGWR Model for BFI

**Fig 7:**
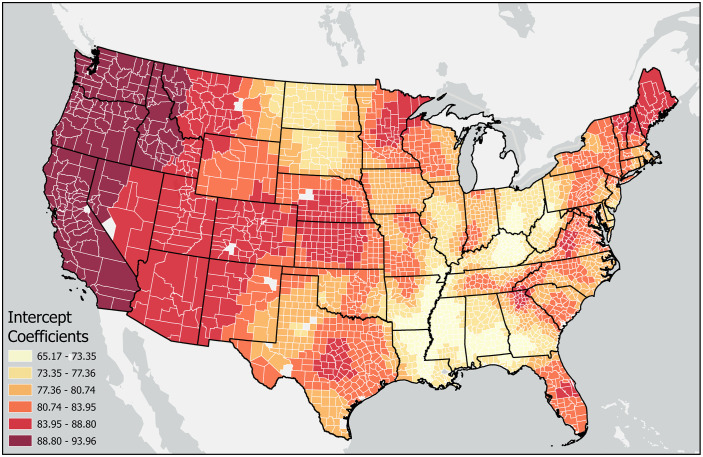
MGWR intercept coefficients representing county-level baseline breastfeeding initiation rates across the contiguous United States, independent of modeled predictors. Basemap Sources: Contains information from OpenStreetMap and OpenStreetMap Foundation, which is made available under the Open Database License.

Fig 7 provides one final snapshot of the MGWR model in action. The model’s local intercepts reveal a persistent regional variation in breastfeeding initiation rates, even after adjusting for the important covariates detailed above, including race, education, income, employment, and urbanity. It is important to remember that the dependent variable remains in its original measurement unit (percent of BFI), while the covariates are standardized. As a result, each local intercept reflects the expected breastfeeding initiation rate for a county when all covariates assume their mean values. Thus, one should consider the intercepts as a way to capture the influence of unmeasured contextual factors such as cultural norms, community support, institutional trust, healthcare infrastructure, or historical patterns of health behavior that help/hinder BFI that vary geographically and are not directly included in the model [46]. Not surprisingly, Fig 7 shows that higher baseline BFI rates concentrate along the Pacific Coast, in parts of the Mountain West, in the Northeast, and in Central Texas. In contrast, lower intercepts cluster in the Gulf Coast region and Appalachia.

## 4. Discussion

Three facets of the results merit additional discussion. First, the results of the MGWR, especially when compared to the simple OLS model, suggest that traditional linear approaches to understanding the spatial distribution of breastfeeding initiation and its drivers may be inadequate for understanding the multiscalar nature of BFI. Specifically, while the Gaussian model was successful in identifying the factors influencing BFI (Table 2), its predictive power was diminished by the underlying (and undetected) spatial dynamics of both the dependent and independent variables. In fact, the OLS model failed to find a significant relationship between BFI and Asian or urban populations. While this failure is not disastrous by any means, these nuances are evident in models that better account for geography and the spatial heterogeneities embedded in both the dependent and independent model variables. In short, the multiscale geographically weighted regression model provided a better fit, was more predictive, and exhibited lower error (Tables 2 and 3).

Second, several facets of the MGWR model can inform policy and planning efforts to improve breastfeeding initiation rates in the United States. For example, we know that several global BFI trends are relatively sticky. In short, *education* and *race* always matter (especially in counties with larger shares of Black residents), as does urbanity. These findings confirm previous work in the field [47–49]. Beyond these drivers of BFI, the MGWR also indicates several regional trends that suggest a more nuanced interpretation of BFI geography in the U.S. is necessary.

For example, there was a strong positive association between BFI and Hispanic populations in the United States, but the MGWR suggests this was primarily in the eastern U.S., not in the West or Southwest, where Hispanic populations are larger. What makes the East Coast special? Several hypotheses present themselves. First, the geographic distribution of Hispanic subgroups differs markedly across the U.S. – the Northeast is home to large Puerto Rican, Dominican, and Central American communities, while Mexican-origin populations predominate in the West and Southwest – and breastfeeding norms and practices vary across these subgroups. Second, acculturation may play a role: a substantial body of research indicates that higher levels of acculturation among Hispanic women are associated with lower breastfeeding rates, with recently arrived and less-acculturated Hispanic immigrants consistently more likely to breastfeed than their U.S.-born counterparts (Bigman et al., 2018). Regional differences in the composition and acculturation levels of Hispanic populations may therefore help explain the east-west gradient in the Hispanic-BFI association observed here. Fig 7, which maps the intercept values, provides additional evidence for pursuing further work on unseen or unmodeled contexts [50].

Fig 5 reveals another example of these geographic differences, highlighting a localized and spatially heterogeneous association between disability prevalence and breastfeeding initiation at the county level. We know that many of the challenges associated with breastfeeding initiation are amplified for mothers with physical disabilities [51–53]. However, the results of the MGWR model suggest that the association between disability prevalence and BFI is sufficiently localized to warrant further investigation into support networks and contextual factors. What are these communities doing differently? Again, more work is necessary. One caveat is that the clusters of BFI-positive locations also occur in areas where breastfeeding initiation lags behind much of the United States (Figs 1 and 5).

The third and final facet of the results that can inform policy and planning efforts seeking to improve breastfeeding initiation rates in the United States is a simple one. Breastfeeding initiation is a local problem. While federal and state efforts to enhance a positive culture for breastfeeding, including workplace accommodations, are beneficial [54,55], challenges for improving BFI remain. Figs 1 and 7 highlight the disparities in breastfeeding initiation, with the latter strongly suggesting local interventions for community-level breastfeeding support and education are necessary to eliminate BFI disparities in the United States. In fact, there are three actionable and cost-effective strategies that have a proven track record for improving BFI. First, communities could enhance access to Baby Friendly Hospitals, which are designed to help mothers to initiate and continue breastfeeding for as long as feasibly possible [56,57]. All Baby-Friendly hospitals have staff, often an international board-certified lactation consultant (IBCLC), to help meet the ten steps to successful breastfeeding (WHO, 2024). Second, communities can work to encourage the establishment of peer-to-peer support (in person and online) [58,59]. Again, this can take the form of community-based support, such as La Leche League or Breastfeeding USA groups, or through online forums for working or more rural/isolated mothers [24]. Third, there is the potential for expanding WIC eligibility [60] and removing income restrictions for mothers seeking breastfeeding support. All of these strategies have the potential to benefit mothers but may be particularly effective in regions with low BFI, such as the Gulf Coast and Appalachia.

Finally, it is important to acknowledge the limitations of our approach and study results. First, many of the usual limitations apply to our study, particularly the exclusion of data (e.g., Michigan), reliance on secondary data (e.g., ACS), and a slight mismatch between BFI and ACS data. However, as detailed previously, the temporal ordering of the BFI and ACS data was required to ensure robust statistical results, and we believe the results of our analysis are likely generalizable to Michigan, given its BFI rates and demographic and socioeconomic profile. We also acknowledge the potential for ecological fallacy. While we focus exclusively on county-level determinants of BFI in this paper, it is important to note that these data do not reflect individual behavior, and there are likely variations in BFI at sub-county levels. Unfortunately, data on individual breastfeeding initiation choices are not widely available for testing.

## 5. Conclusion

Breastfeeding confers substantial health benefits to both mother and child. While these benefits are generally well-known, breastfeeding initiation rates in the United States are highly variable. In this paper, we use multiscale geographically weighted regression, controlling for a range of demographic and socioeconomic variables, to model BFI rates. Our spatial statistical approach highlighted that race, education, and urbanity are always important for understanding BFI rates, but that distinct pockets of spatial heterogeneity in BFI also dot the landscape, where other drivers (e.g., disability, female-headed households, and females in the labor force) are similarly important. Identifying these geographical nuances provides valuable guidance for identifying opportunities to enhance community-level breastfeeding support (e.g., La Leche League), healthcare infrastructure (e.g., Baby Friendly Hospitals), and education efforts (WIC). Specifically, many communities across Appalachia and the Gulf Coast lag behind California, the Pacific Northwest, and the Northeast in breastfeeding initiation rates, but cost-effective and directed intervention efforts in these lagging communities may yield long-term benefits to mothers and infants and move the United States closer to its Healthy People 2030 goals for breastfeeding initiation.

## References

[1] Chowdhury R, Sinha B, Sankar MJ, Taneja S, Bhandari N, Rollins N, et al. Breastfeeding and maternal health outcomes: A systematic review and meta-analysis. Acta Paediatr. 2015;104(467):96–113. doi: 10.1111/apa.13102 26172878 PMC4670483

[2] Neville CE, McKinley MC, Holmes VA, Spence D, Woodside JV. The relationship between breastfeeding and postpartum weight change--A systematic review and critical evaluation. Int J Obes (Lond). 2014;38(4):577–90. doi: 10.1038/ijo.2013.132 23892523

[3] McGowan C, Bland R. The benefits of breastfeeding on child intelligence, behavior, and executive function: A review of recent evidence. Breastfeeding Medicine. 2023;18:172–87. doi: 10.1089/bfm.2022.0192 36749962

[4] Peres KG, Cascaes AM, Nascimento GG, Victora CG. Effect of breastfeeding on malocclusions: A systematic review and meta-analysis. Acta Paediatr. 2015;104(467):54–61. doi: 10.1111/apa.13103 26140303

[5] Horta BL, de Lima NP. Breastfeeding and type 2 diabetes: Systematic review and meta-analysis. Curr Diab Rep. 2019;19(1):1. doi: 10.1007/s11892-019-1121-x 30637535

[6] Hernández‐Cordero S, Pérez‐Escamilla R. What will it take to increase breastfeeding?. Maternal & Child Nutrition. 2022;18:e13371. doi: 10.1111/mcn.13371 PMC9113470 35534910

[7] Ip S, Chung M, Raman G, Chew P, Magula N, DeVine D, et al. Breastfeeding and maternal and infant health outcomes in developed countries. Evid Rep Technol Assess (Full Rep). 2007;(153):1–186. 17764214 PMC4781366

[8] Sankar MJ, Sinha B, Chowdhury R, Bhandari N, Taneja S, Martines J, et al. Optimal breastfeeding practices and infant and child mortality: A systematic review and meta-analysis. Acta Paediatr. 2015;104(467):3–13. doi: 10.1111/apa.13147 26249674

[9] CDC. About breastfeeding data. Centers for Disease Control and Prevention. https://www.cdc.gov/breastfeeding-data/about/index.html

[10] U.S. DHHS. Healthy People 2030. Department of Health and Human Services. 2021. https://odphp.health.gov/healthypeople

[11] CDC. U.S. birth certificate breastfeeding initiation data, 2018-2019. Centers for Disease Control and Prevention. 2024. https://www.cdc.gov/breastfeeding-data/county-initiation/index.html#

[12] Fotheringham AS, Yang W, Kang W. Multiscale Geographically Weighted Regression (MGWR). Annals of the American Association of Geographers. 2017;107(6):1247–65. doi: 10.1080/24694452.2017.1352480

[13] Wang F, Zhang T, Zhang S. MGWR reveals scale heterogeneity shaping intangible cultural heritage distribution in China. npj Herit Sci. 2025;13(1). doi: 10.1038/s40494-025-01938-x

[14] Quintero SM, Strassle PD, Londoño Tobón A, Ponce S, Alhomsi A, Maldonado AI, et al. Race/ethnicity-specific associations between breastfeeding information source and breastfeeding rates among U.S. women. BMC Public Health. 2023;23(1):520. doi: 10.1186/s12889-023-15447-8 36932332 PMC10024358

[15] Crowell A, Fossett M. Metropolitan racial residential segregation in the United States: A microlevel and cross-context analysis of Black, Latino, and Asian segregation. Demographic Research. 2022;46:217–60. doi: 10.4054/DemRes.2022.46.8

[16] Pérez-Escamilla R, Martinez JL, Segura-Pérez S. Impact of the baby-friendly hospital Initiative on breastfeeding and child health outcomes: A systematic review. Matern Child Nutr. 2016;12(3):402–17. doi: 10.1111/mcn.12294 26924775 PMC6860129

[17] Walsh A, Pieterse P, Mishra N, Chirwa E, Chikalipo M, Msowoya C, et al. Improving breastfeeding support through the implementation of the Baby-Friendly Hospital and Community Initiatives: A scoping review. Int Breastfeed J. 2023;18(1):22. doi: 10.1186/s13006-023-00556-2 37061737 PMC10105160

[18] Grubesic TH, Durbin KM. Geodemographies of breastfeeding support. J Hum Lact. 2021;37(2):301–13. doi: 10.1177/0890334420941416 32735482

[19] Gross SM, Lerman JL, Hurley KM, Venkataramani M, Sharma R, Ogunwole SM, et al. Breastfeeding outcomes associated with the special supplemental nutrition program for women, infants, and children: A systematic review. Acad Pediatr. 2023;23(2):244–60. doi: 10.1016/j.acap.2022.10.008 36272723

[20] Chetwynd EM, Wasser HM, Poole C. Breastfeeding support interventions by international board certified lactation consultants: A systemic review and meta-analysis. J Hum Lact. 2019;35(3):424–40. doi: 10.1177/0890334419851482 31206317

[21] Grubesic TH, Durbin KM. Breastfeeding support: A geographic perspective on access and equity. J Hum Lact. 2017;33(4):770–80. doi: 10.1177/0890334417706361 28602120

[22] Grubesic TH, Durbin KM. A spatial analysis of breastfeeding and breastfeeding support in the United States: The leaders and laggards landscape. J Hum Lact. 2019;35(4):790–800. doi: 10.1177/0890334419856615 31206311

[23] Chua CMS, Mathews J, Ong MSB, Liew KK, Shorey S. Use of telelactation interventions to improve breastfeeding outcomes among mothers: A mixed-studies systematic review. Women Birth. 2023;36(3):247–56. doi: 10.1016/j.wombi.2022.06.011 35792035

[24] Grubesic TH, Durbin KM. The complex geographies of telelactation and access to community breastfeeding support in the state of Ohio. PLoS One. 2020;15(11):e0242457. doi: 10.1371/journal.pone.0242457 33232335 PMC7685454

[25] Grubesic TH, Durbin KM. Community rates of breastfeeding initiation: A geospatial analysis of Kentucky. J Hum Lact. 2016;32:601–10. doi: 10.1177/0890334416651070 27502514

[26] Grubesic TH, Durbin KM. Breastfeeding, community vulnerability, resilience, and disasters: A snapshot of the United States Gulf Coast. Int J Environ Res Public Health. 2022;19:11847. doi: 10.3390/ijerph191911847 36231150 PMC9564847

[27] Whitley MD, Ro A, Palma A. Work, race and breastfeeding outcomes for mothers in the United States. PLoS One. 2021;16(5):e0251125. doi: 10.1371/journal.pone.0251125 33951094 PMC8099119

[28] Foster SF, Vazquez C, Cubbin C, Nichols AR, Rickman RR, Widen EM. Breastfeeding, socioeconomic status, and long-term postpartum weight retention. Int Breastfeed J. 2023;18(1):1. doi: 10.1186/s13006-022-00534-0 36600252 PMC9814482

[29] Standish KR, Parker MG. Social determinants of breastfeeding in the United States. Clin Ther. 2022;44(2):186–92. doi: 10.1016/j.clinthera.2021.11.010 34906370

[30] Segura-Pérez S, Hromi-Fiedler A, Adnew M, Nyhan K, Pérez-Escamilla R. Impact of breastfeeding interventions among United States minority women on breastfeeding outcomes: A systematic review. Int J Equity Health. 2021;20(1):72. doi: 10.1186/s12939-021-01388-4 33676506 PMC7936442

[31] Strid P, Simeone RM, Hall R, Meeker JR, Ellington SR. A new tool for estimating the number of pregnant people in the United States. Obstet Gynecol. 2025;145(1):e11–3. doi: 10.1097/AOG.0000000000005750 39361960

[32] Census. Census Quick Facts. https://www.census.gov/quickfacts/fact/table/US/PST045221. 2022.

[33] CDPH. In-Hospital Breastfeeding Initiation Data. California Department of Public Health; 2019. https://www.cdph.ca.gov/Programs/CFH/DMCAH/Breastfeeding/Pages/In-Hospital-Breastfeeding-Initiation-Data.aspx

[34] MDHHS. Breastfeeding Report. MichiganDepartment of Health and Human Services; 2019. https://www.michigan.gov/mdhhs/assistance-programs/wic/datasystems/datareports/specialreports/breastfeeding

[35] Sparks PJ. Rural-urban differences in breastfeeding initiation in the United States. J Hum Lact. 2010;26(2):118–29. doi: 10.1177/0890334409352854 20032310

[36] Tebeje TM, Seifu BL, Mare KU, Asgedom YS, Asmare ZA, Asebe HA, et al. Geospatial determinants and spatio-temporal variation of early initiation of breastfeeding and exclusive breastfeeding in Ethiopia from 2011 to 2019, a multiscale geographically weighted regression analysis. BMC Public Health. 2024;24(1):2011. doi: 10.1186/s12889-024-19552-0 39068397 PMC11282616

[37] Fotheringham AS, Yang W, Kang W. Multiscale Geographically Weighted Regression (MGWR). Annals of the American Association of Geographers. 2017;107(6):1247–65. doi: 10.1080/24694452.2017.1352480

[38] Fotheringham AS, Oshan TM, Li Z. Multiscale Geographically Weighted Regression: Theory and Practice. 1st ed. Boca Raton: CRC Press. 2023. doi: 10.1201/9781003435464

[39] Anselin L, Sridharan S, Gholston S. Using exploratory spatial data analysis to leverage social indicator databases: The discovery of interesting patterns. Soc Indic Res. 2007;82:287–309. doi: 10.1007/s11205-006-9034-x

[40] Tobler WR. A computer movie simulating urban growth in the Detroit Region. Economic Geography. 1970;46:234. doi: 10.2307/143141

[41] Brunsdon C, Fotheringham S, Charlton M. Geographically weighted regression. J Royal Statistical Soc D. 1998;47:431–43. doi: 10.1111/1467-9884.00145

[42] Beale CM, Lennon JJ, Yearsley JM, Brewer MJ, Elston DA. Regression analysis of spatial data. Ecol Lett. 2010;13(2):246–64. doi: 10.1111/j.1461-0248.2009.01422.x 20102373

[43] Yu H, Fotheringham AS, Li Z, Oshan T, Kang W, Wolf LJ. Inference in multiscale geographically weighted regression. Geographical Analysis. 2019;52(1):87–106. doi: 10.1111/gean.12189

[44] Shabrina Z, Buyuklieva B, Ng MKM. Short‐term rental platform in the urban tourism context: A Geographically Weighted Regression (GWR) and a Multiscale GWR (MGWR) Approaches. Geographical Analysis. 2020;53(4):686–707. doi: 10.1111/gean.12259

[45] Webster GR, Bowman J. Quantitatively delineating the black belt Geographic Region. sgo. 2008;48(1):3–18. doi: 10.1353/sgo.0.0007

[46] Fotheringham AS, Li Z. Measuring the unmeasurable: Models of geographical context. Annals of the American Association of Geographers. 2023;113(10):2269–86. doi: 10.1080/24694452.2023.2227690

[47] Kirksey K. A social history of racial disparities in breastfeeding in the United States. Soc Sci Med. 2021;289:114365. doi: 10.1016/j.socscimed.2021.114365 34592542

[48] Li R, Perrine CG, Anstey EH, Chen J, MacGowan CA, Elam-Evans LD. Breastfeeding Trends by Race/Ethnicity among US children born from 2009 to 2015. JAMA Pediatr. 2019;173(12):e193319. doi: 10.1001/jamapediatrics.2019.3319 31609438 PMC6802058

[49] Petit M, Smart DA, Sattler V, Wood NK. Examination of factors that contribute to breastfeeding disparities and inequities for black women in the US. J Nutr Educ Behav. 2021;53(11):977–86. doi: 10.1016/j.jneb.2021.08.013 34763821

[50] Kwan MP. The uncertain geographic context problem. Annals of the Association of American Geographers. 2012;102:958–68. doi: 10.1080/00045608.2012.687349

[51] Andrews EE, Powell RM, Ayers KB. Experiences of breastfeeding among disabled women. Womens Health Issues. 2021;31(1):82–9. doi: 10.1016/j.whi.2020.09.001 33051056 PMC9531779

[52] Brown HK, Pablo L, Scime NV, Aker AM, Dennis C-L. Maternal disability and initiation and duration of breastfeeding: analysis of a Canadian cross-sectional survey. Int Breastfeed J. 2023;18(1):70. doi: 10.1186/s13006-023-00608-7 38129879 PMC10734132

[53] Powell RM, Mitra M, Smeltzer SC, Long-Bellil LM, Smith LD, Rosenthal E, et al. Breastfeeding among women with physical disabilities in the United States. J Hum Lact. 2018;34(2):253–61. doi: 10.1177/0890334417739836 29166569

[54] Chang Y-S, Harger L, Beake S, Bick D. Women’s and Employers’ Experiences and views of combining breastfeeding with a return to paid employment: A systematic review of qualitative studies. J Midwifery Womens Health. 2021;66(5):641–55. doi: 10.1111/jmwh.13243 34423557

[55] Harrigan PB, Schenk T, Volpe SL, Hedrick VE, Khan T, Misyak SA. A comparison of U.S. infant feeding policy changes to Global Breastfeeding Collective policy priorities. Front Public Health. 2025;13:1653377. doi: 10.3389/fpubh.2025.1653377 41041368 PMC12484190

[56] Fan YW, Fan HSL, Shing JSY, Ip HL, Fong DYT, Lok KYW. Impact of baby-friendly hospital initiatives on breastfeeding outcomes: Systematic review and meta-analysis. Women Birth. 2025;38(2):101881. doi: 10.1016/j.wombi.2025.101881 39919651

[57] Shing JS, Lok KY, Fong DY, Fan HS, Chow CL, Tarrant M. The influence of the baby-friendly hospital initiative and maternity care practices on breastfeeding outcomes. J Hum Lact. 2022;38(4):700–10. doi: 10.1177/08903344221086975 35403491

[58] Moon H, Woo K. An integrative review on mothers’ experiences of online breastfeeding peer support: Motivations, attributes and effects. Matern Child Nutr. 2021;17(3):e13200. doi: 10.1111/mcn.13200 33960665 PMC8189189

[59] Yang Y, Liu H, Cui X, Meng J. Mothers’ experiences and perceptions of breastfeeding peer support: A qualitative systematic review. Int Breastfeed J. 2024;19(1):7. doi: 10.1186/s13006-024-00614-3 38243287 PMC10797811

[60] Buxbaum SG, Arigbede O, Mathis A, Close F, Suther SG, Mazzio E, et al. Disparities in infant nutrition: WIC participation and rates of breastfeeding in Florida. Int J Environ Res Public Health. 2023;20(11):5988. doi: 10.3390/ijerph20115988 37297592 PMC10253221

